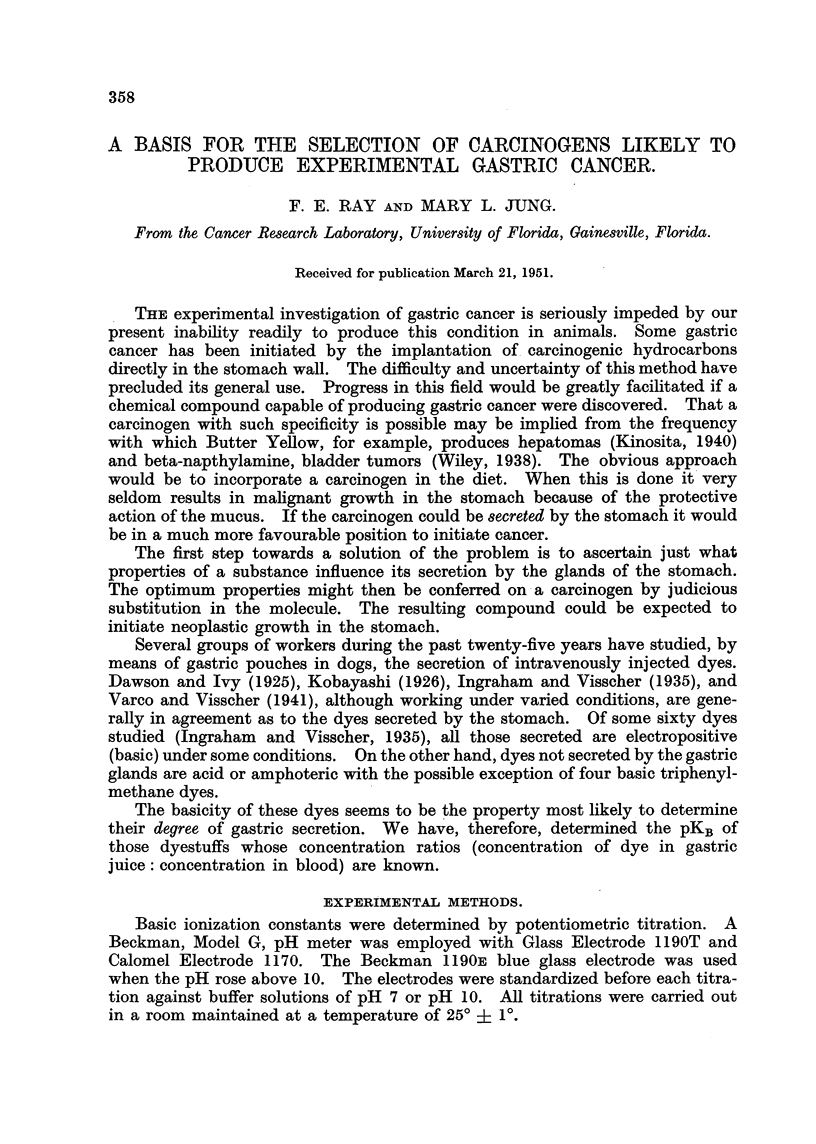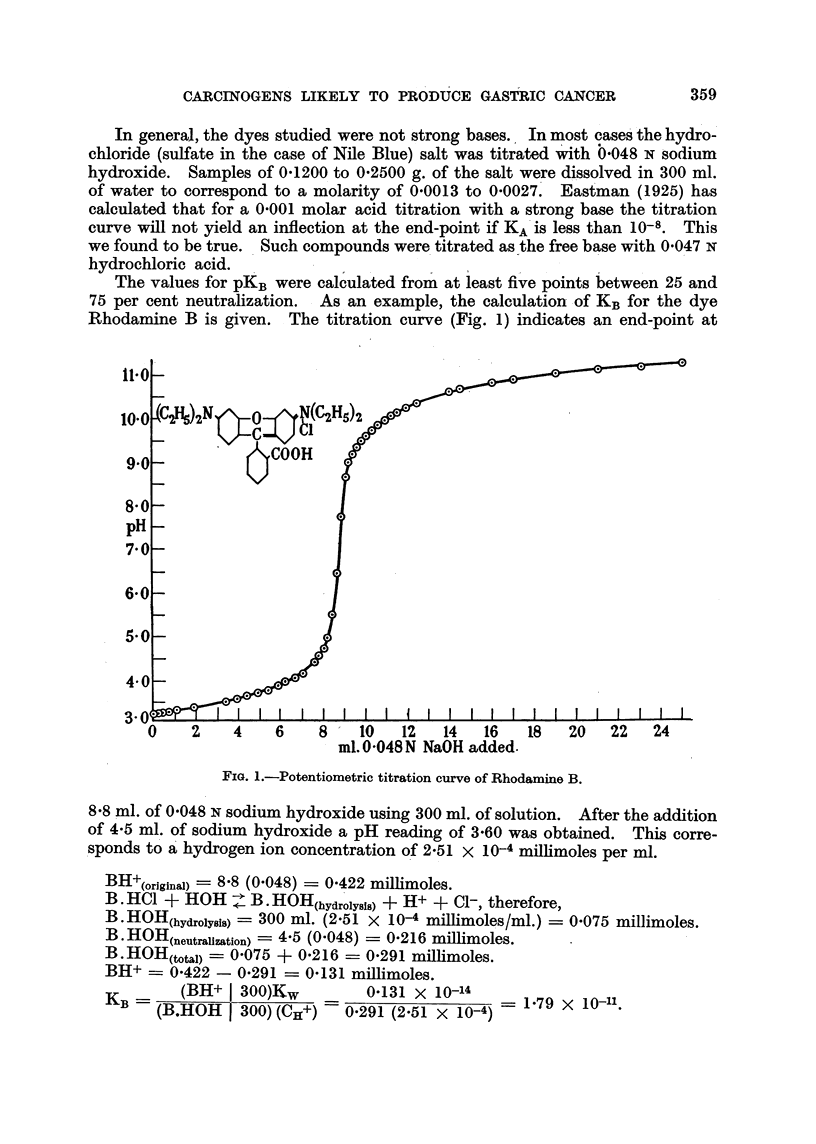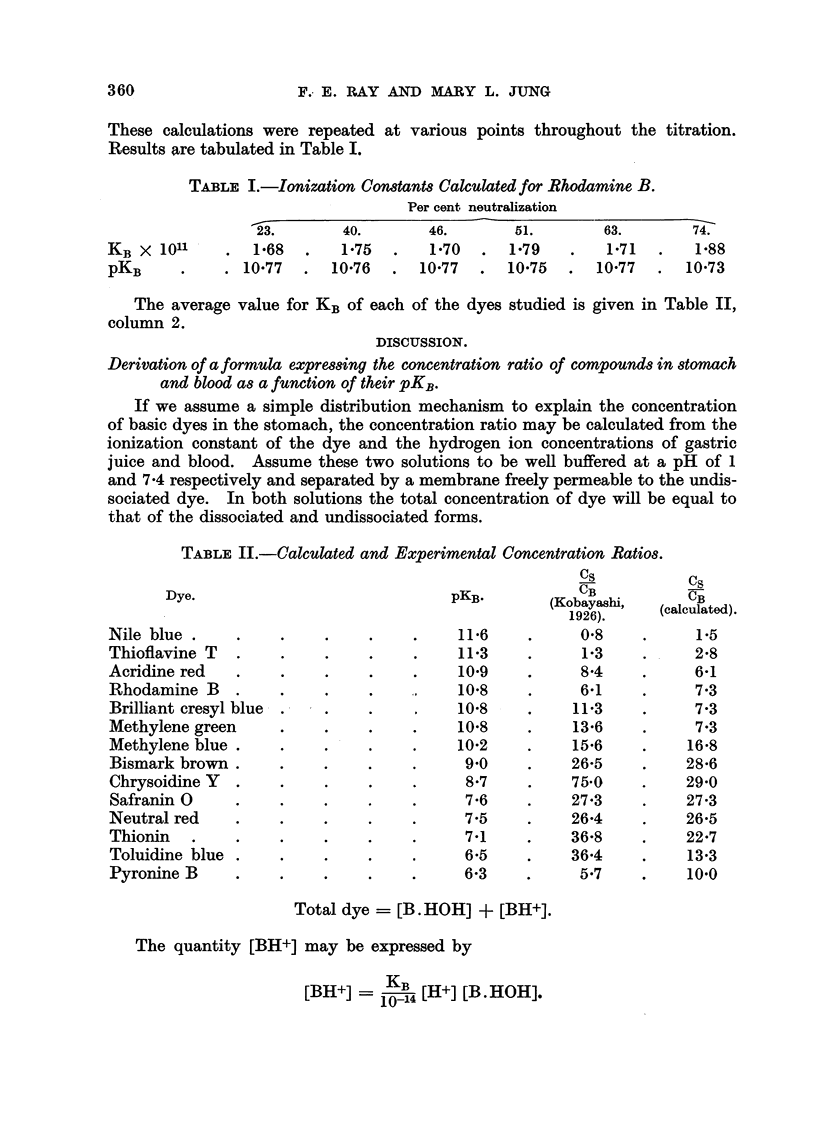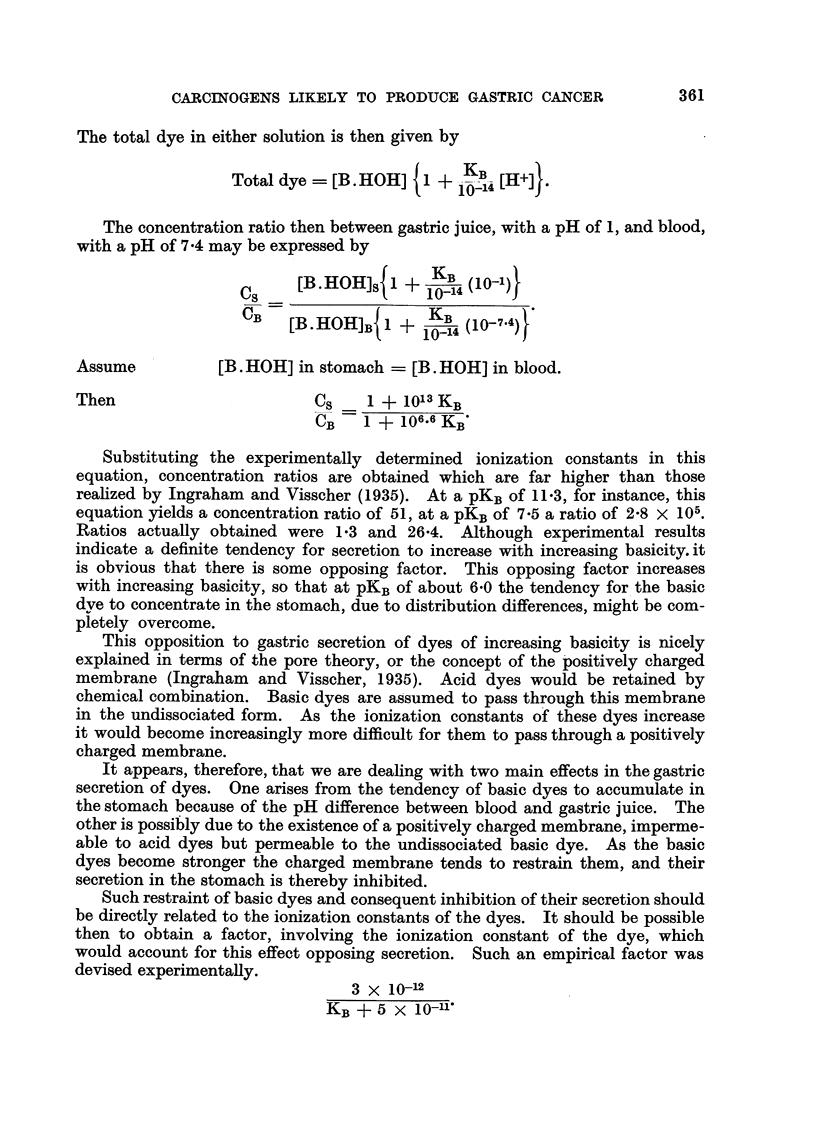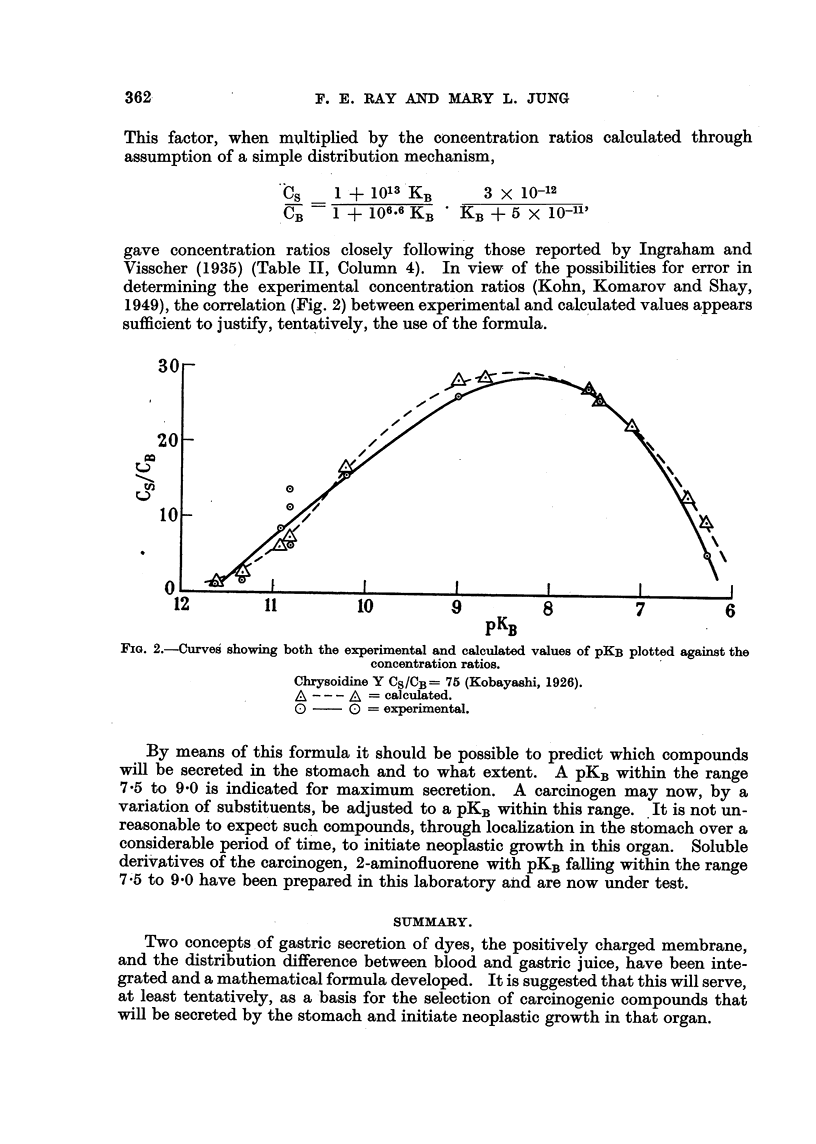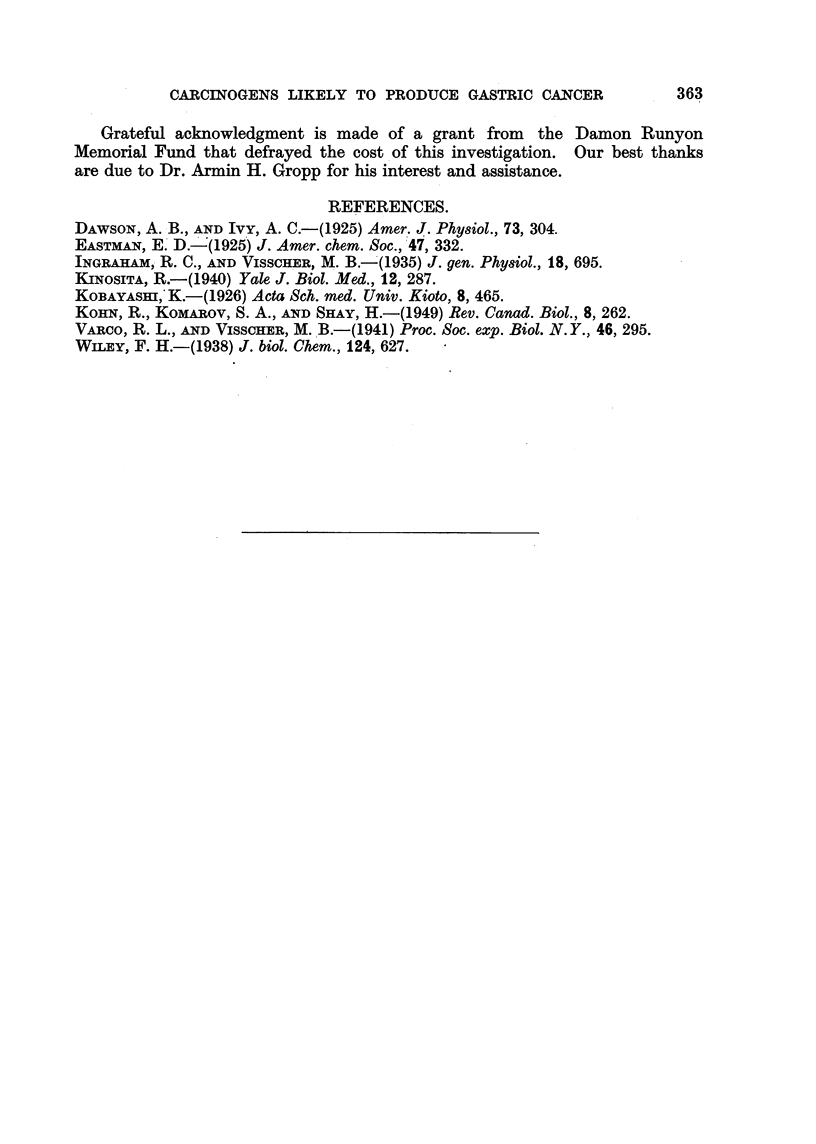# A Basis for the Selection of Carcinogens likely to Produce Experimental Gastric Cancer

**DOI:** 10.1038/bjc.1951.38

**Published:** 1951-09

**Authors:** F. E. Ray, Mary L. Jung


### A BASIS FOR THE SELECTION OF CARCINOGENS LIKELY TO PRODUCE EXPERIMENTAL GASTRIC CANCER.

F. E. RAY AND MARY L. JUNG.

*From the Cancer Research Laboratory, University of Florida, Gainesville, Florida.*

Received for publication March 21, 1951.

THE experimental investigation of gastric cancer is seriously impeded by our present inability readily to produce this condition in animals. Some gastric cancer has been initiated by the implantation of carcinogenic hydrocarbons directly in the stomach wall. The difficulty and uncertainty of this method have precluded its general use. Progress in this field would be greatly facilitated if a chemical compound capable of producing gastric cancer were discovered. That a carcinogen with such specificity is possible may be implied from the frequency with which Butter Yellow, for example, produces hepatomas (Kinosita, 1940) and beta-napthylamine, bladder tumors (Wiley, 1938). The obvious approach would be to incorporate a carcinogen in the diet. When this is done it very seldom results in malignant growth in the stomach because of the protective action of the mucus. If the carcinogen could be *secreted* by the stomach it would be in a much more favourable position to initiate cancer.

The first step towards a solution of the problem is to ascertain just what properties of a substance influence its secretion by the glands of the stomach. The optimum properties might then be conferred on a carcinogen by judicious substitution in the molecule. The resulting compound could be expected to initiate neoplastic growth in the stomach.

Several groups of workers during the past twenty-five years have studied, by means of gastric pouches in dogs, the secretion of intravenously injected dyes. Dawson and Ivy (1925), Kobayashi (1926), Ingraham and Visscher (1935), and Varco and Visscher (1941), although working under varied conditions, are generally in agreement as to the dyes secreted by the stomach. Of some sixty dyes studied (Ingraham and Visscher, 1935), all those secreted are electropositive (basic) under some conditions. On the other hand, dyes not secreted by the gastric glands are acid or amphoteric with the possible exception of four basic triphenylmethane dyes.

The basicity of these dyes seems to be the property most likely to determine their *degree* of gastric secretion. We have, therefore, determined the $pK_B$ of those dyestuffs whose concentration ratios (concentration of dye in gastric juice : concentration in blood) are known.

### EXPERIMENTAL METHODS.

Basic ionization constants were determined by potentiometric titration. A Beckman, Model G, pH meter was employed with Glass Electrode 1190T and Calomel Electrode 1170. The Beckman 1190E blue glass electrode was used when the pH rose above 10. The electrodes were standardized before each titration against buffer solutions of pH 7 or pH 10. All titrations were carried out in a room maintained at a temperature of 25° $\pm$ 1°.

In general, the dyes studied were not strong bases. In most cases the hydrochloride (sulfate in the case of Nile Blue) salt was titrated with 0·048 N sodium hydroxide. Samples of 0·1200 to 0·2500 g. of the salt were dissolved in 300 ml. of water to correspond to a molarity of 0·0013 to 0·0027. Eastman (1925) has calculated that for a 0·001 molar acid titration with a strong base the titration curve will not yield an inflection at the end-point if $K_A$ is less than $10^{-8}$. This we found to be true. Such compounds were titrated as the free base with 0·047 N hydrochloric acid.

The values for $pK_B$ were calculated from at least five points between 25 and 75 per cent neutralization. As an example, the calculation of $K_B$ for the dye Rhodamine B is given. The titration curve (Fig. 1) indicates an end-point at

FIG. 1.—Potentiometric titration curve of Rhodamine B.

8·8 ml. of 0·048 N sodium hydroxide using 300 ml. of solution. After the addition of 4·5 ml. of sodium hydroxide a pH reading of 3·60 was obtained. This corresponds to a hydrogen ion concentration of $2 \cdot 51 \times 10^{-4}$ millimoles per ml.

$BH^{+}_{(original)} = 8 \cdot 8\ (0 \cdot 048) = 0 \cdot 422$ millimoles.

$B.HCl + HOH \rightleftarrows B.HOH_{(hydrolysis)} + H^{+} + Cl^{-}$, therefore,

$B.HOH_{(hydrolysis)} = 300$ ml. $(2 \cdot 51 \times 10^{-4}$ millimoles/ml.$) = 0 \cdot 075$ millimoles.

$B.HOH_{(neutralization)} = 4 \cdot 5\ (0 \cdot 048) = 0 \cdot 216$ millimoles.

$B.HOH_{(total)} = 0 \cdot 075 + 0 \cdot 216 = 0 \cdot 291$ millimoles.

$BH^{+} = 0 \cdot 422 - 0 \cdot 291 = 0 \cdot 131$ millimoles.

$$K_B = \frac{(BH^{+} \mid 300)K_W}{(B.HOH \mid 300)\,(C_{H^{+}})} = \frac{0 \cdot 131 \times 10^{-14}}{0 \cdot 291\ (2 \cdot 51 \times 10^{-4})} = 1 \cdot 79 \times 10^{-11}.$$

These calculations were repeated at various points throughout the titration. Results are tabulated in Table I.

TABLE I.—*Ionization Constants Calculated for Rhodamine B.*

| | Per cent neutralization | | | | | |
|---|---|---|---|---|---|---|
| | 23. | 40. | 46. | 51. | 63. | 74. |
| $K_B \times 10^{11}$ | 1·68 | 1·75 | 1·70 | 1·79 | 1·71 | 1·88 |
| $pK_B$ | 10·77 | 10·76 | 10·77 | 10·75 | 10·77 | 10·73 |

The average value for $K_B$ of each of the dyes studied is given in Table II, column 2.

### DISCUSSION.

*Derivation of a formula expressing the concentration ratio of compounds in stomach and blood as a function of their $pK_B$.*

If we assume a simple distribution mechanism to explain the concentration of basic dyes in the stomach, the concentration ratio may be calculated from the ionization constant of the dye and the hydrogen ion concentrations of gastric juice and blood. Assume these two solutions to be well buffered at a pH of 1 and 7·4 respectively and separated by a membrane freely permeable to the undissociated dye. In both solutions the total concentration of dye will be equal to that of the dissociated and undissociated forms.

TABLE II.—*Calculated and Experimental Concentration Ratios.*

| Dye. | $pK_B$. | $\frac{C_S}{C_B}$ (Kobayashi, 1926). | $\frac{C_S}{C_B}$ (calculated). |
|---|---|---|---|
| Nile blue | 11·6 | 0·8 | 1·5 |
| Thioflavine T | 11·3 | 1·3 | 2·8 |
| Acridine red | 10·9 | 8·4 | 6·1 |
| Rhodamine B | 10·8 | 6·1 | 7·3 |
| Brilliant cresyl blue | 10·8 | 11·3 | 7·3 |
| Methylene green | 10·8 | 13·6 | 7·3 |
| Methylene blue | 10·2 | 15·6 | 16·8 |
| Bismark brown | 9·0 | 26·5 | 28·6 |
| Chrysoidine Y | 8·7 | 75·0 | 29·0 |
| Safranin O | 7·6 | 27·3 | 27·3 |
| Neutral red | 7·5 | 26·4 | 26·5 |
| Thionin | 7·1 | 36·8 | 22·7 |
| Toluidine blue | 6·5 | 36·4 | 13·3 |
| Pyronine B | 6·3 | 5·7 | 10·0 |

$$\text{Total dye} = [\text{B.HOH}] + [\text{BH}^+].$$

The quantity $[\text{BH}^+]$ may be expressed by

$$[\text{BH}^+] = \frac{K_B}{10^{-14}} [\text{H}^+] [\text{B.HOH}].$$

The total dye in either solution is then given by

$$\text{Total dye} = [\text{B.HOH}]\left\{1 + \frac{K_B}{10^{-14}}[H^+]\right\}.$$

The concentration ratio then between gastric juice, with a pH of 1, and blood, with a pH of 7·4 may be expressed by

$$\frac{C_S}{C_B} = \frac{[\text{B.HOH}]_S\left\{1 + \frac{K_B}{10^{-14}}(10^{-1})\right\}}{[\text{B.HOH}]_B\left\{1 + \frac{K_B}{10^{-14}}(10^{-7.4})\right\}}.$$

Assume [B.HOH] in stomach = [B.HOH] in blood.

Then

$$\frac{C_S}{C_B} = \frac{1 + 10^{13} K_B}{1 + 10^{6.6} K_B}.$$

Substituting the experimentally determined ionization constants in this equation, concentration ratios are obtained which are far higher than those realized by Ingraham and Visscher (1935). At a $pK_B$ of 11·3, for instance, this equation yields a concentration ratio of 51, at a $pK_B$ of 7·5 a ratio of $2{\cdot}8 \times 10^5$. Ratios actually obtained were 1·3 and 26·4. Although experimental results indicate a definite tendency for secretion to increase with increasing basicity. it is obvious that there is some opposing factor. This opposing factor increases with increasing basicity, so that at $pK_B$ of about 6·0 the tendency for the basic dye to concentrate in the stomach, due to distribution differences, might be completely overcome.

This opposition to gastric secretion of dyes of increasing basicity is nicely explained in terms of the pore theory, or the concept of the positively charged membrane (Ingraham and Visscher, 1935). Acid dyes would be retained by chemical combination. Basic dyes are assumed to pass through this membrane in the undissociated form. As the ionization constants of these dyes increase it would become increasingly more difficult for them to pass through a positively charged membrane.

It appears, therefore, that we are dealing with two main effects in the gastric secretion of dyes. One arises from the tendency of basic dyes to accumulate in the stomach because of the pH difference between blood and gastric juice. The other is possibly due to the existence of a positively charged membrane, impermeable to acid dyes but permeable to the undissociated basic dye. As the basic dyes become stronger the charged membrane tends to restrain them, and their secretion in the stomach is thereby inhibited.

Such restraint of basic dyes and consequent inhibition of their secretion should be directly related to the ionization constants of the dyes. It should be possible then to obtain a factor, involving the ionization constant of the dye, which would account for this effect opposing secretion. Such an empirical factor was devised experimentally.

$$\frac{3 \times 10^{-12}}{K_B + 5 \times 10^{-11}}.$$

This factor, when multiplied by the concentration ratios calculated through assumption of a simple distribution mechanism,

$$\frac{C_S}{C_B} = \frac{1 + 10^{13} K_B}{1 + 10^{6.6} K_B} \cdot \frac{3 \times 10^{-12}}{K_B + 5 \times 10^{-11}},$$

gave concentration ratios closely following those reported by Ingraham and Visscher (1935) (Table II, Column 4). In view of the possibilities for error in determining the experimental concentration ratios (Kohn, Komarov and Shay, 1949), the correlation (Fig. 2) between experimental and calculated values appears sufficient to justify, tentatively, the use of the formula.

FIG. 2.—Curves showing both the experimental and calculated values of $pK_B$ plotted against the concentration ratios.

Chrysoidine Y $C_S/C_B$ = 75 (Kobayashi, 1926).

△ - - - △ = calculated.

⊙ —— ⊙ = experimental.

By means of this formula it should be possible to predict which compounds will be secreted in the stomach and to what extent. A $pK_B$ within the range 7·5 to 9·0 is indicated for maximum secretion. A carcinogen may now, by a variation of substituents, be adjusted to a $pK_B$ within this range. It is not unreasonable to expect such compounds, through localization in the stomach over a considerable period of time, to initiate neoplastic growth in this organ. Soluble derivatives of the carcinogen, 2-aminofluorene with $pK_B$ falling within the range 7·5 to 9·0 have been prepared in this laboratory and are now under test.

SUMMARY.

Two concepts of gastric secretion of dyes, the positively charged membrane, and the distribution difference between blood and gastric juice, have been integrated and a mathematical formula developed. It is suggested that this will serve, at least tentatively, as a basis for the selection of carcinogenic compounds that will be secreted by the stomach and initiate neoplastic growth in that organ.

Grateful acknowledgment is made of a grant from the Damon Runyon Memorial Fund that defrayed the cost of this investigation. Our best thanks are due to Dr. Armin H. Gropp for his interest and assistance.

### REFERENCES.

Dawson, A. B., and Ivy, A. C.—(1925) *Amer. J. Physiol.*, **73**, 304.

Eastman, E. D.—(1925) *J. Amer. chem. Soc.*, **47**, 332.

Ingraham, R. C., and Visscher, M. B.—(1935) *J. gen. Physiol.*, **18**, 695.

Kinosita, R.—(1940) *Yale J. Biol. Med.*, **12**, 287.

Kobayashi, K.—(1926) *Acta Sch. med. Univ. Kioto*, **8**, 465.

Kohn, R., Komarov, S. A., and Shay, H.—(1949) *Rev. Canad. Biol.*, **8**, 262.

Varco, R. L., and Visscher, M. B.—(1941) *Proc. Soc. exp. Biol. N.Y.*, **46**, 295.

Wiley, F. H.—(1938) *J. biol. Chem.*, **124**, 627.